# Convertible ROS Nanocatalyst/Eliminator for Enhancing Tumor Radio‐Immunotherapy and Relieving Radiation Enteritis

**DOI:** 10.1002/advs.202503602

**Published:** 2025-06-10

**Authors:** Mengmeng Zhang, Xinyu Zhang, Chenyu Wang, Mengyao Mu, Ke Ren, Nengyi Ni, Li Xian Yip, Kai Guo, Xiaoyu Hu, Feifei Teng, David Tai Leong, Qing Fan, Xiao Sun

**Affiliations:** ^1^ Department of Pharmacy Shandong Cancer Hospital and Institute Shandong First Medical University & Shandong Academy of Medical Sciences Jinan 250117 China; ^2^ Medical Science and Technology Innovation Center Tumor Research and Therapy Center Shandong Provincial Hospital Affiliated to Shandong First Medical University Jinan 250021 China; ^3^ Department of Chemical and Biomolecular Engineering National University of Singapore Singapore 117585 Singapore

**Keywords:** efficacy enhancement, Janus nanoarchitectures, radiation enteritis, radiotherapy, toxicity reduction

## Abstract

Radiation‐induced damage to normal tissue seriously disrupts the execution of radiotherapy (RT) and therefore attenuates its efficacy. Exploring new strategies to enhance the efficacy and reduce toxicity of RT has great clinical importance. Here, a PD‐L1 affibody (Z_PD‐L1_) grafted Janus Ag/Ag_2_S nanoarchitectures is developed as “reactive oxygen species lever” to enhance radiocatalysis and relieve radiation enteritis. The nanoplatform can achieve abundant energy deposition in the form of high‐energy electrons within tumors upon X‐ray exposure. Subsequently, the transfer of electrons from Ag_2_S to Ag moiety enables effective electron–hole pair separation, which greatly improves the radiocatalysis efficiency in generating highly toxic hydroxyl radical (•OH). Due to the specific binding to tumor PD‐L1, Z_PD‐L1_ endows this nanoplatform with efficient tumor targeting and meanwhile reduces immune suppression of T cells by achieving PD‐1/PD‐L1 blockade, intensifying RT‐induced immunologic cell death. Notably, the presence of the Ag moiety allows the nanoplatform to act as an H_2_O_2_ scavenger in intestinal tissues during metabolism, effectively relieving radiation enteritis. Moreover, systemic administration of the nanoplatform coupled with Mn‐DOTA can guide RT timing selection by magnetic resonance imaging. Overall, this nanoplatform can substantially improve RT effectiveness with reduced radiation enteritis, demonstrating considerable potential in tumor RT.

## Graphical abstract

A novel Janus Ag/Ag_2_S nanostructure grafted with dimer PD‐L1 affibody (AMZ) is successfully developed. AMZ can be used as ROS nanocatalyst to achieve tumor radiosensitization, and it can also act as an ROS eliminator to alleviate radiation enteritis, achieving a radiotherapy strategy characterized by “toxicity reduction and efficacy enhancement”.

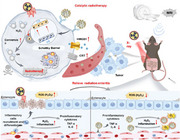

## 1 Introduction

Radiotherapy (RT) stands as a prominent method for cancer treatment, especially playing a significant role in the treatment of abdominal tumors. It is capable of eliminating tumor cells through both direct nuclear DNA damage caused by high‐energy X‐rays and indirect injury induced by reactive oxygen species (ROS).^[^ ^1^, ^2^ ^]^ However, the intrinsic radioresistance of tumor cells weakens the efficacy of RT.^[^ ^3^, ^4^ ^]^ Additionally, RT faces the historic challenge of dosage, where the use of high radiation doses to effectively kill tumor cells will inevitably cause collateral damage to surrounding normal tissues, with radiation enteritis emerging as a common complication. Research indicates that the incidence of radiation enteritis in patients undergoing abdominal RT can exceed 40%. Moreover, current RT management systems lack proactive mechanisms to address the issue of radiation enteritis, and available anti‐inflammatory strategies are mostly passive responses activated after inflammation occurs, typically involving symptomatic drug treatment. In some cases, this may even necessitate the termination of RT to manage severe inflammatory responses. These factors can significantly disrupt the application of RT, greatly attenuating its efficacy. Therefore, exploring new strategies to enhance the efficacy and reduce toxicity of RT has great clinical significance.

Catalytic radiotherapy, an emerging chemical enhancement strategy in tumor treatment, involves designing and constructing nanocatalysts to increase the production of X‐ray‐induced hydroxyl radicals (•OH).^[^ ^5^, ^6^, ^7^ ^]^ It is worth noting that the overexpressed H_2_O_2_ in the tumor microenvironment provided a natural molecular target for catalytic radiotherapy.^[^ ^8^ ^]^ Semiconductor nanomaterials, with their unique band structures (conduction band (CB) and valence band (VB)), offer inherent advantages in generating radicals. For instance, some semiconductor nanomaterials can catalyze H_2_O or hydrogen peroxide (H_2_O_2_) into •OH under light irradiation.^[^ ^9^, ^10^ ^]^ However, the bandgap of semiconductor nanomaterials is usually narrow, which can easily cause the rapid recombination of photogenerated electron–hole pairs after separation, limiting the generation efficiency of •OH. Furthermore, the combination of semiconductor nanomaterials with noble metals has been shown to effectively promote the transfer and rearrangement of electrons at the interface by forming Schottky barriers, thus significantly enhancing the catalytic generation efficiency of •OH.^[^ ^11^, ^12^, ^13^, ^14^ ^]^ The Janus heterojunction, in particular, exhibits enhanced catalytic properties through interface interaction, making it a highly effective radiosensitizer. They can rapidly convert overexpressed H_2_O_2_ in tumors into large amounts of •OH via a catalytic reaction triggered by X‐rays.^[^ ^15^, ^16^ ^]^

Additionally, the overproduction of ROS during RT is one of the key factors in driving the intestinal inflammatory microenvironment.^[^ ^17^ ^]^ Despite the relatively short X‐ray irradiation duration, the activated immune cells and proinflammatory mediators significantly enhance H_2_O_2_ production in normal tissues, creating a positive feedback loop that disrupts the redox balance.^[^ ^18^ ^]^ Therefore, it is essential to restore the redox homeostasis of inflammatory tissues by eliminating ROS. In recent years, various noble metal‐based catalase‐like nanozymes containing high atomic number (Z) elements, such as Ag, have garnered considerable attention.^[^ ^19^, ^20^ ^]^ Studies have shown that Ag can consume H_2_O_2_ and release O_2_.^[^ ^21^ ^]^ Based on this, introducing Ag into Janus heterojunction holds promise as an ideal “ROS lever”, enabling the generation of a large amount of ROS in tumors under the stimulation of RT, while also possessing the significant potential to eliminate ROS in normal tissues to reduce inflammation. Moreover, ionizing radiation in RT not only directly kills cancer cells but also triggers immunogenic cell death (ICD), leading to the release of damage‐associated molecular patterns (DAMPs), promoting dendritic cell maturation, and activating anti‐tumor immune responses.^[^ ^22^ ^]^ Despite these benefits, it cannot be ignored that RT can induce tumor cells to overexpress PD‐L1 and reduce the anti‐tumor effect of T cells. Combining PD‐L1 inhibitors to block the PD‐L1 immune checkpoint was an effective strategy to overcome RT‐induced immune evasion.^[^ ^23^ ^]^

**Scheme 1:**
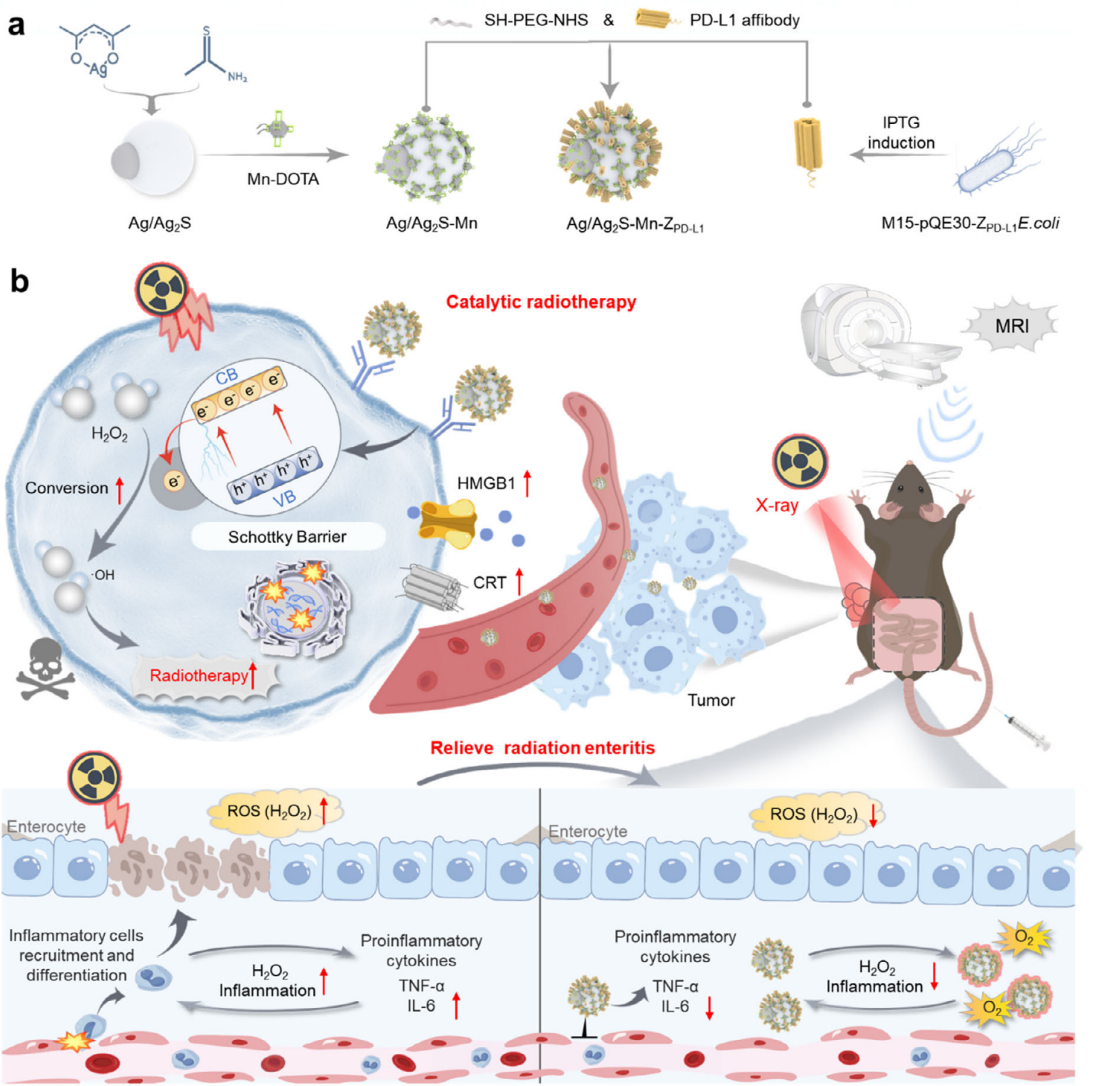
a) Schematic illustration of the synthesis of AMZ, and b) its mechanism for enhancing the radiocatalysis efficiency to generate highly toxic •OH under X‐ray exposure.

Here, PD‐L1 affibodies (Z_PD‐L1_) grafted Janus Ag/Ag_2_S nanoarchitectures (AMZ) were designed as ROS levers to enhance radiocatalysis and relieve radiation enteritis (**Scheme** **1**). Upon X‐ray exposure, the nanoplatform could deposit high energy in tumors with the form of high‐energy electrons. The Schottky barrier between Ag and Ag_2_S facilitated efficient electron transfer, enabling the separation of X‐ray‐triggered electron–hole pairs and significantly enhancing the production efficiency of •OH within tumors. The modification with Z_PD‐L1_ not only granted the nanoplatform strong tumor targeting ability but also effectively blocked the PD‐1/PD‐L1 interaction, further reversing immune T‐cell suppression and amplifying RT‐induced ICD. The presence of Ag moieties enabled the nanoplatform to serve as an H_2_O_2_ scavenger in intestinal tissues during metabolism, effectively relieving radiation enteritis. Additionally, the nanoplatform could utilize MRI to optimize the radiosensitization timing. Overall, this nanoplatform successfully achieved efficient radiosensitivity with enhanced immune regulation, and relief of radiation enteritis, offering a promising catalytic radiotherapy approach.

## 2 Results and Discussion

### 2.1 Synthesis and Characterization

**Figure 1:**
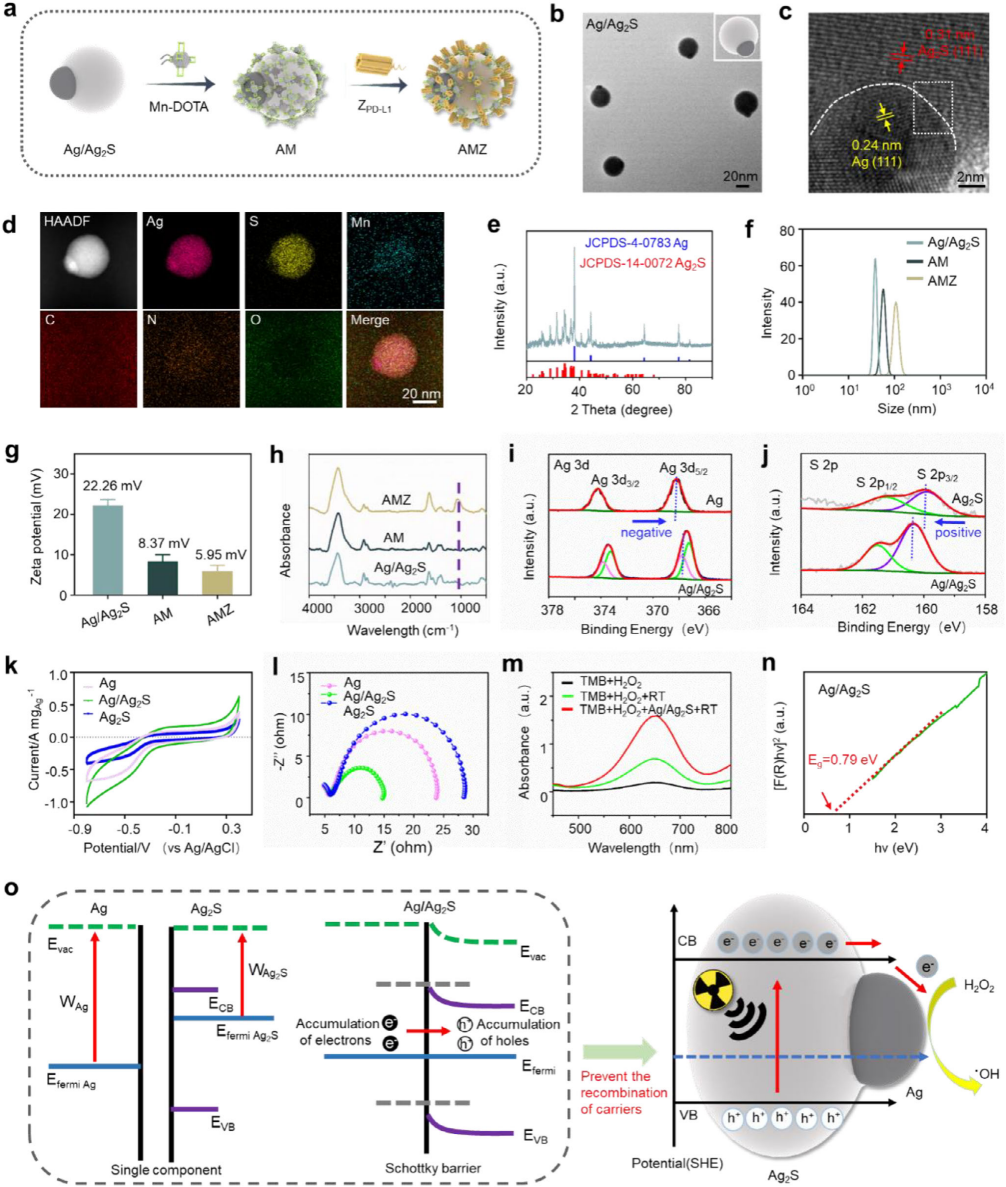
a) Schematic illustration of the synthesis of AMZ. b) TEM image of Ag/Ag_2_S. c) HR‐TEM images of Ag/Ag_2_S. d) Element mapping images of AMZ. e) XRD of Ag/Ag_2_S. f) DLS analysis, g) Zeta potential analysis, and h) FTIR spectra of AMZ. i) XPS spectra of Ag 3d signals of Ag and Ag/Ag_2_S. j) XPS spectra of S 2p signals of Ag_2_S and Ag/Ag_2_S. k) Cyclic voltammetry curves of Ag, Ag/Ag_2_S and Ag_2_S. l) EIS Nyquist plot of Ag, Ag/Ag_2_S and Ag_2_S. m) The UV–vis spectra of TMB solution under different treatments. n) Energy bandgaps of Ag/Ag_2_S. o) Plausible mechanism for the enhanced •OH generation through X‐ray‐induced catalytic process in Schottky heterostructures of Ag/Ag_2_S.

The synthesis of AMZ was carried out through a three‐step process, as depicted in **Figure** **1**a. Initially, Ag/Ag_2_S with a Janus structure was prepared using a modified one‐pot method, employing Ag(acac)_2_ as the Ag source and thioacetamide (TAA) as the sulfur source. Subsequently, Mn‐DOTA was grafted onto the Ag/Ag_2_S precursor to form Ag/Ag_2_S‐Mn (AM). Finally, Z_PD‐L1_ affibody was loaded onto the surface of AM to prepare AMZ. The formation of Janus Ag/Ag_2_S occurred in two independent phases. Transmission electron microscopy (TEM) image revealed that the particles exhibited a uniform Janus morphology, with good dispersion and an average size of ≈40 nm (Figure 1b). The darker region was identified as Ag, and the asymmetric morphology was attributed to the presence of Ag_2_S. High‐resolution TEM (HR‐TEM) image clearly showed independent lattice fringes of the two components, with lattice spacings of 0.24 nm corresponding to the cubic Ag (111) plane and 0.31 nm to the monoclinic Ag_2_S (111) plane (Figure 1c). Additionally, the HR‐TEM image exhibited an atomically smooth interface and connected lattice between Ag_2_S and Ag, indicating the formation of Schottky heterojunctions through crosslinking of Ag_2_S and Ag, rather than being physically mixed or isolated. X‐ray diffraction patterns (XRD) confirmed the successful synthesis of Ag/Ag_2_S, with diffraction peak positions and relative intensities matching those of monoclinic Ag_2_S and cubic Ag (Figure 1e).

Furthermore, the TEM image demonstrated that the morphology and composition of AMZ remained unchanged after grafting Mn‐DOTA and Z_PD‐L1_ onto the surface of Ag/Ag_2_S (Figure S1, Supporting Information). Elemental mapping revealed a uniform distribution of Ag, S, Mn, C, N, and O elements, confirming the successful synthesis of AMZ (Figure 1d). Dynamic light scattering (DLS) results showed a slight increase in the nanoparticle size during the synthesis of Ag/Ag_2_S, AM and AMZ (Figure 1f). This was accompanied by a corresponding decrease in the zeta potential: from 22.26 mV (Ag/Ag_2_S), 8.37 mV (AM) to 5.95 mV (AMZ) (Figure 1g), validating the successful completion of each synthetic step. Fourier‐transform infrared spectroscopy (FTIR) identified several new peaks in AMZ compared to Ag/Ag_2_S, including the characteristic peaks at 1050 cm^−1^ attributed to the amide bonds associated with protein‐peptide groups of Z_PD‐L1_, further confirming the successful preparation of AMZ (Figure 1h). Additionally, to ensure the long‐term stability of AMZ during systemic administration, a 7‐day in vitro dynamic stability monitoring was conducted. The results indicated that during the incubation period, AMZ showed no obvious size changes, and neither the loaded Z_PD‐L1_ nor Mn ions were released, verifying the excellent stability of AMZ (Figures S2 and S3, Supporting Information).

### 2.2 Exploration of Radiocatalysis Activity

X‐ray photoelectron spectroscopy (XPS) was employed to investigate the electron states of as‐prepared Janus Ag/Ag_2_S. For better comparison, pure Ag and Ag_2_S were also investigated. Compared with the binding energies (BEs) of Ag 3d_5/2_ and Ag 3d_3/2_ signals of pure Ag (368.2 and 374.2 eV), those of Ag/Ag_2_S (367.8 and 373.8 eV) were negatively shifted, indicating that the Ag portion of Ag/Ag_2_S had a higher electron density (Figure 1i).^[^ ^24^, ^25^ ^]^ However, the BEs of S 2p_3/2_ and S 2p_1/2_ signals of Ag/Ag_2_S (160.3 and 161.5 eV) were positively shifted compared with those of Ag_2_S (160.0 and 161.2 eV), suggesting a decrease in electron density in the Ag_2_S portion of Ag/Ag_2_S due to electron loss (Figure 1j).^[^ ^26^ ^]^ The differences in BEs of Ag and S in Janus Ag/Ag_2_S heterostructure indicated that electrons were transferred from Ag_2_S to Ag, resulting in negative charge accumulation on the Ag surface. To further investigate the electron transfer at the interface of Janus Ag/Ag_2_S, we examined the photocurrent responses of as‐prepared samples under X‐ray irradiation, as this served as direct evidence of enhanced electron–hole pairs generation at the Schottky heterojunction.^[^ ^14^ ^]^ As shown in Figure 1k, Ag/Ag_2_S exhibited a better photocurrent response than a single component under X‐ray irradiation. Furthermore, the electrochemical impedance spectroscopy (EIS) spectrum revealed that Ag/Ag_2_S had the smallest Nyquist circle radius, indicating the lowest charge transfer resistance (Figure 1l). These findings suggested that the Schottky heterostructure formed by coupling Ag with Ag_2_S can effectively facilitate the separation of electrons and holes triggered by X‐rays, which was crucial for the generation of •OH. Compared to conditions without Ag/Ag_2_S, the degradation rate of TMB by Ag/Ag_2_S after X‐ray irradiation was close to 73.5% (Figure 1m). Since the enhancement of TMB degradation was primarily due to the increased production of •OH, these results indicated that Ag/Ag_2_S can enhance energy deposition under irradiation, thereby effectively accelerating the decomposition of overexpressed H_2_O_2_ in tumor microenvironment into highly toxic •OH through the X‐ray‐induced catalytic reaction.

According to the absorption spectra of Ag/Ag_2_S and Ag_2_S converted by UV–vis diffuse reflection spectra (Figures S4 and S5, Supporting Information), the bandgap value of Ag/Ag_2_S was calculated to be 0.79 eV, which was significantly lower than Ag_2_S (1.03 eV) (Figure 1n; Figure S6, Supporting Information). The results indicated that the introduction of Ag reduced the bandgap of the Ag_2_S semiconductor, thereby enhancing its ability to capture long‐wavelength photons and improving the efficiency of excited electron–hole separation. Based on these findings, a plausible mechanism for the enhanced generation •OH by Ag/Ag_2_S was presented (Figure 1o; Figure S7, Supporting Information). This enhancement was attributed to the formation of the Schottky barrier between Ag_2_S and Ag, and when electrons in the VB were excited to the CB by X‐rays, the Schottky barrier trapped the electrons and transferred them to Ag. This process thereby prevented the recombination of electron–hole pairs, allowing H_2_O_2_, which acted as an electron acceptor, to decompose into toxic •OH that kills tumor cells. Taken together, these results confirmed that Ag/Ag_2_S can serve as an effective radiation electron collector of •OH production, thereby improving RT efficacy.

### 2.3 In Vitro Antitumor Effect of AMZ

**Figure 2:**
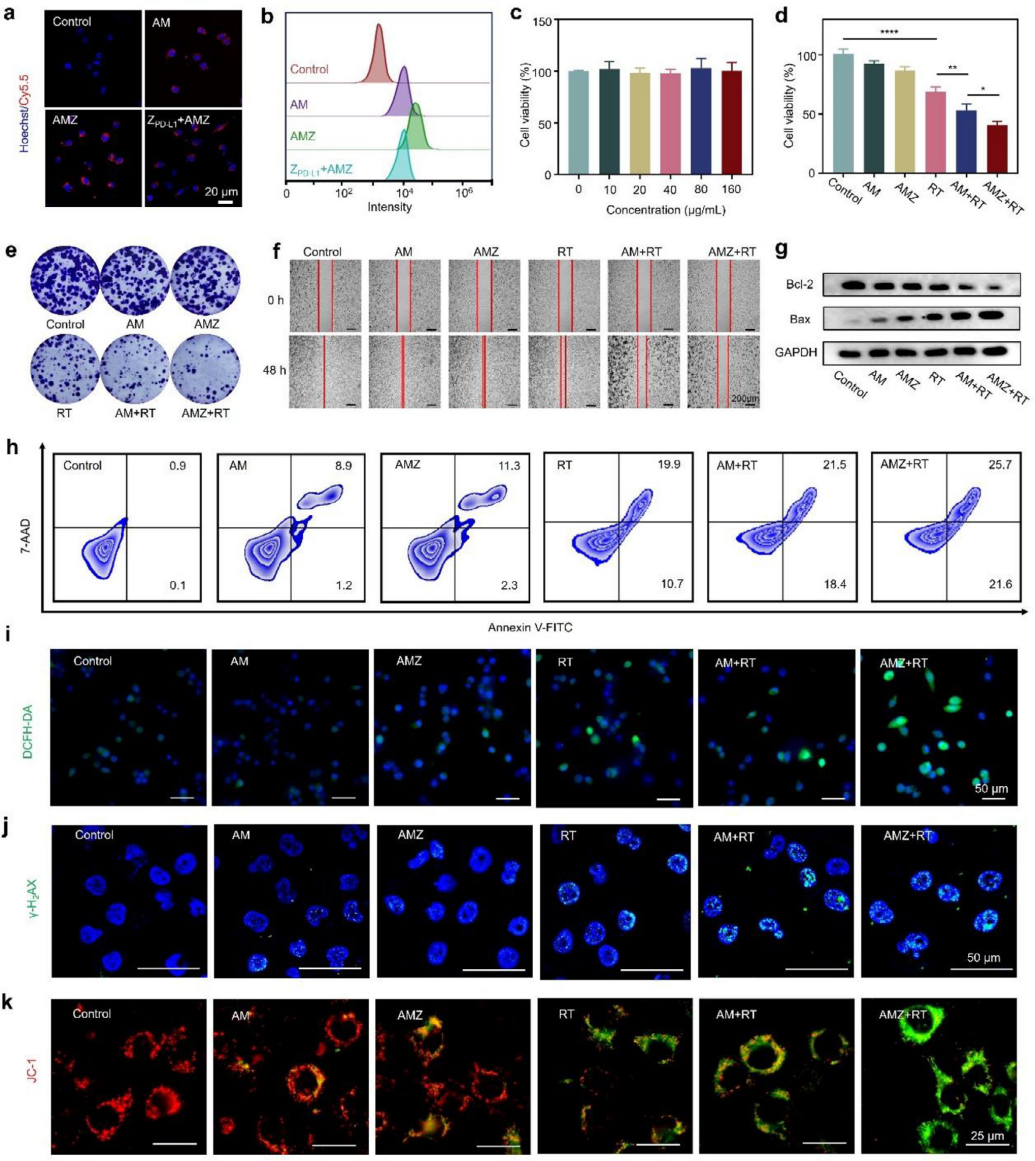
a) CLSM images of MC38 cells with different treatments. b) FCM analysis of MC38 cells with different treatments. c) Biocompatibility evaluation of HUVEC with different concentrations (*n* = 5). d) MTT assays of MC38 cells with different treatments (*n* = 5). e) Clone formation images of MC38 cells with different treatments. f) Wound healing images of MC38 cells with different treatments. g) WB analysis of MC38 cells with different treatments. h) FCM of apoptosis in MC38 cells with different treatments. i) Fluorescence images of MC38 cells stained with DCFH‐DA. j) Fluorescence images of MC38 cells stained with γ‐H2AX. k) Fluorescence images of MC38 cells stained with JC‐1. Statistical analyses were determined by one‐way ANOVA. ^*^ *p* < 0.05, ^**^ *p* < 0.01, ^****^ *p* < 0.0001.

The cellular uptake experiments were conducted using confocal laser scanning microscopy (CLSM) and flow cytometry (FCM). MC38 cells treated with AMZ exhibited significantly enhanced red fluorescence compared to the AM group, indicating improved tumor targeting mediated by Z_PD‐L1C_ (**Figure** **2**a; Figure S8, Supporting Information). FCM analysis displayed a similar trend to that observed in CLSM (Figure 2b; Figure S9, Supporting Information), which further demonstrated the efficient cellular uptake performance of AMZ. When MC38 cells were pretreated with Z_PD‐L1_, the red fluorescence signals were notably reduced, suggesting that partial blocking of PD‐L1 receptors on MC38 cells limited Z_PD‐L1_‐mediated tumor targeting, providing additional evidence for the successful conjugation of Z_PD‐L1_ to AMZ. The biocompatibility of AMZ was further assessed, and the results showed that the cell viability of HUVEC remained above 95% within the range of AMZ treatment concentrations given, indicating the good compatibility of AMZ (Figure 2c).

Furthermore, the in vitro efficacy of RT was evaluated. As shown in Figure 2d, AMZ combined with X‐ray (AMZ+RT) exerted the most pronounced inhibitory effect on MC38 cells compared to other groups. This may be due to the highly efficient catalytic activity of AMZ, which can enhance X‐ray damage to tumor cells, thus achieving effective radiosensitization. Colony formation experiments revealed severe suppression of MC38 cell colony formation in the AMZ+RT group, underscoring the promising potential of AMZ as an effective radiosensitizer (Figure 2e; Figure S10, Supporting Information). In addition, wound healing assays showed the slowest healing rate for MC38 cells in the AMZ+RT group, extending up to 48 h (Figure 2f; Figure S11, Supporting Information). These results demonstrated the highly effective radiosensitization of AMZ.

Subsequently, the radiosensitization mechanism of AMZ was further explored. Western blotting (WB) assays showed that the expression of pro‐apoptotic protein Bax was highest in the AMZ+RT group, while the expression of anti‐apoptotic protein Bcl‐2 was lowest. This finding reflected that AMZ significantly enhanced RT‐induced cell apoptosis (Figure 2g; Figure S12, Supporting Information). FCM analysis further confirmed that the apoptosis rate of the AMZ+RT group was significantly higher than other treatment groups (Figure 2h; Figure S13, Supporting Information). Given that X‐ray irradiation typically induced apoptosis by increasing intracellular levels of •OH, we assessed •OH production using DCFH‐DA. As expected, the AMZ+RT group exhibited the highest levels of intracellular •OH compared to other groups (Figure 2i; Figure S14, Supporting Information). Elevated •OH levels were known to induce substantial cellular damage, particularly DNA double‐strand breaks (DSBs), which were hazardous forms of damage in RT. To quantify DNA DSBs, γ‐H2AX foci were detected in cell nuclei via CLSM. The results showed a significant increase in the number of γ‐H2AX foci in AMZ+RT, with 3.2 times more than observed in the RT group (Figure 2j; Figure S15, Supporting Information). This result verified that AMZ+RT induced more severe DNA damage due to the efficient •OH generation via the Schottky barrier. In addition, we assessed mitochondrial membrane potential loss, which serves as an indicator of cellular damage caused by •OH. Consistent with previous results, the AMZ+RT group showed the most significant mitochondrial damage (Figure 2k; Figure S16, Supporting Information). In summary, these results suggested that AMZ demonstrated a strong radiosensitization effect.

Tumor cell death triggered specific molecular events, including the exposure of intracellular calreticulin (CRT) and the release of high mobility group protein B1 (HMGB1). These events activated ICD and stimulated an antitumor immune response. Compared with other groups, CRT exposure was significantly increased in the AMZ+RT group (Figure S17, Supporting Information). Furthermore, the AMZ+RT group exhibited the lowest HMGB1 fluorescence signal in the nuclei of MC38 cells (Figure S18, Supporting Information). These findings suggested that the combination of AMZ and RT effectively induces ICD, promoting dendritic cell maturation and activating cytotoxic T cells.

### 2.4 MRI Performance of AMZ

**Figure 3:**
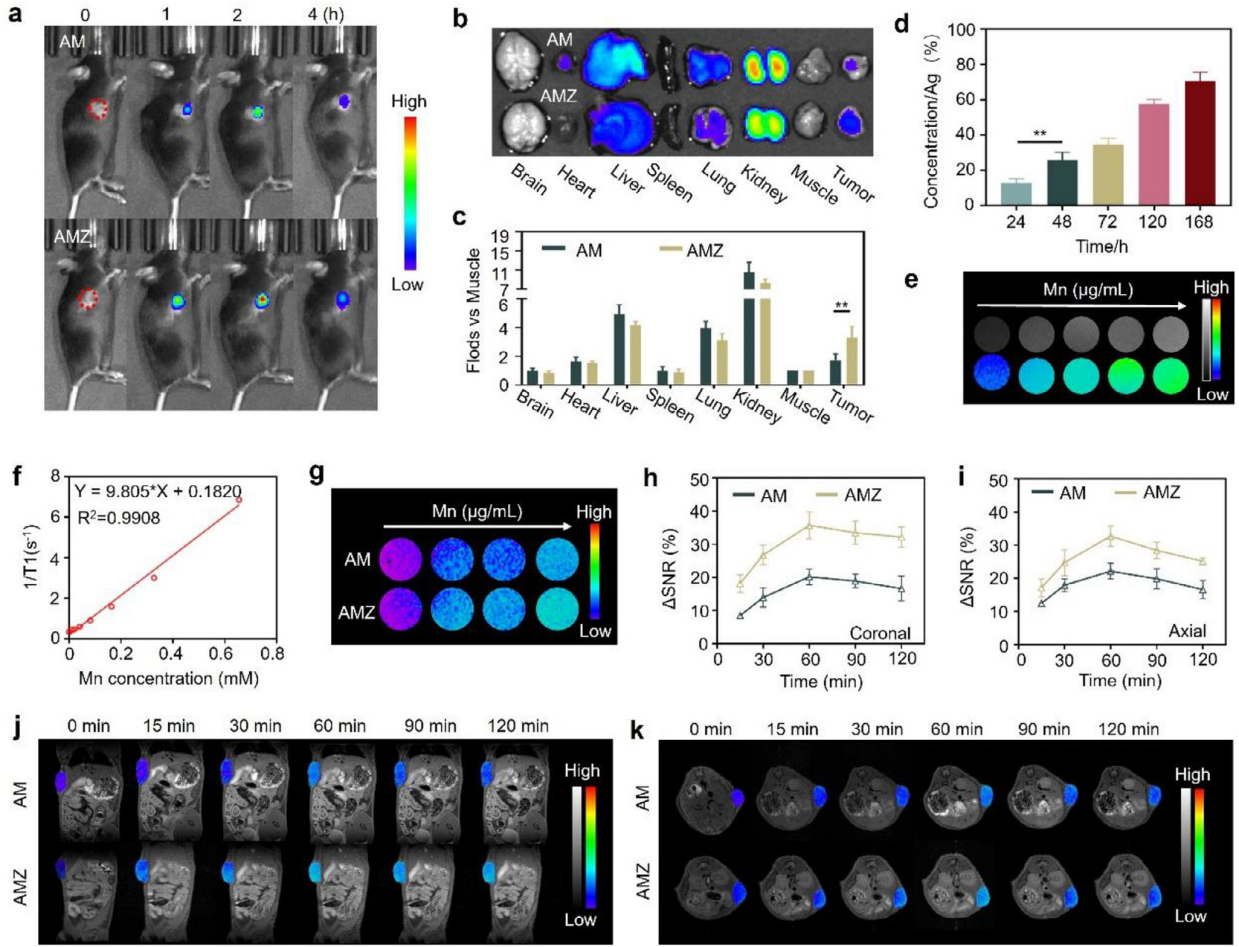
a) Fluorescence distribution of mice at various time points post‐administration of Cy5.5‐labeled AMZ. b) Fluorescence images and c) the corresponding quantitative analysis of isolated organs after 4 h of administration (*n* = 3). d) Cumulative percentage of Ag in feces collected at different time points after tail vein injection of AMZ (*n* = 3). e)T1‐weighted MRI and f) longitudinal relaxation rate of AMZ solution. g) T1‐weighted MRI of MC38 cells after different treatments. j) T1‐weighted MRI of coronal positions of mice acquired at different post‐injection times of AM and AMZ, and h) the corresponding ΔSNR. k) T1‐weighted MRI of axial positions of mice acquired at different post‐injection times of AM and AMZ, and i) the corresponding ΔSNR. Statistical analyses were determined by one‐way ANOVA. ^**^ *p* < 0.01.

The accumulation of AMZ in tumors was investigated using fluorescence imaging of mice. Compared to the AM group, tumor tissues in the AMZ group exhibited a significant fluorescence enhancement in tumor‐bearing areas, indicating that AMZ could target tumors more effectively (**Figure** **3**a). Furthermore, in the dissected organs or tissues of the mice, the AMZ group displayed increased fluorescence intensity in tumor tissues relative to the AM group, further confirming the favorable targeting ability of AMZ for tumors (Figure 3b,c). These findings demonstrated that AMZ could effectively accumulate in tumor tissues for in vivo MRI and radiosensitization. In addition, the concentration of Ag in feces was measured by ICP‐OES. The results showed that within 168 h, most of the Ag from AMZ was metabolized via feces (Figure 3d), demonstrating the good in vivo biosafety of AMZ. The pharmacokinetic results further corroborated the high metabolic capacity of AMZ (Figure S19, Supporting Information).

MRI was commonly regarded as the gold standard for evaluating the extent of tumor invasion and diagnosing its boundaries.^[^ ^27^, ^28^, ^29^ ^]^ MRI‐guided RT has facilitated the precise targeting of radiation doses in target areas, optimizing the choice of RT time to obtain the best RT efficacy.^[^ ^30^ ^]^ As shown in Figure 3e, the AMZ solution exhibited Mn^2+^ concentration‐dependent MRI enhancement. The longitudinal relaxation rate (r1) of AMZ was calculated to be 9.805 mM^−1^ s^−1^, almost 2.88 times higher than commercial MRI contrast agents (r1 ≈ 3.40 mM^−1^ s^−1^),^[^ ^31^ ^]^ indicating superior MRI performance of AMZ (Figure 3f). Furthermore, AMZ‐treated MC38 cells showed better MRI performance than AM at the same Mn^2+^ concentration (Figure 3g), suggesting that AMZ improved tumor targeting capabilities.

Subsequently, the MRI performance of AMZ in vivo was further evaluated. Both coronal and axial T1‐weighted images (Figure 3j,k) of tumor sites in mice showed gradually increased MRI signals and then decreased over time, with the peak occurring at 60 min post‐injection, indicating enrichment of the nanoplatform within the tumor and providing guidance for the timing of subsequent in vivo RT. The calculated signal‐to‐noise ratio (ΔSNR) for AMZ was 35.2±2.1% and 32.7±1.8% (Figure 3h), much higher than that of the AM group, which had a peak ΔSNR of 19.7±1.2% and 22.3±1.2% (Figure 3i). These results suggested that AMZ exhibited a higher and faster tumor enrichment capacity, leading to a stronger MRI signal in tumors.

### 2.5 In Vivo Antitumor Effect of AMZ

**Figure 4:**
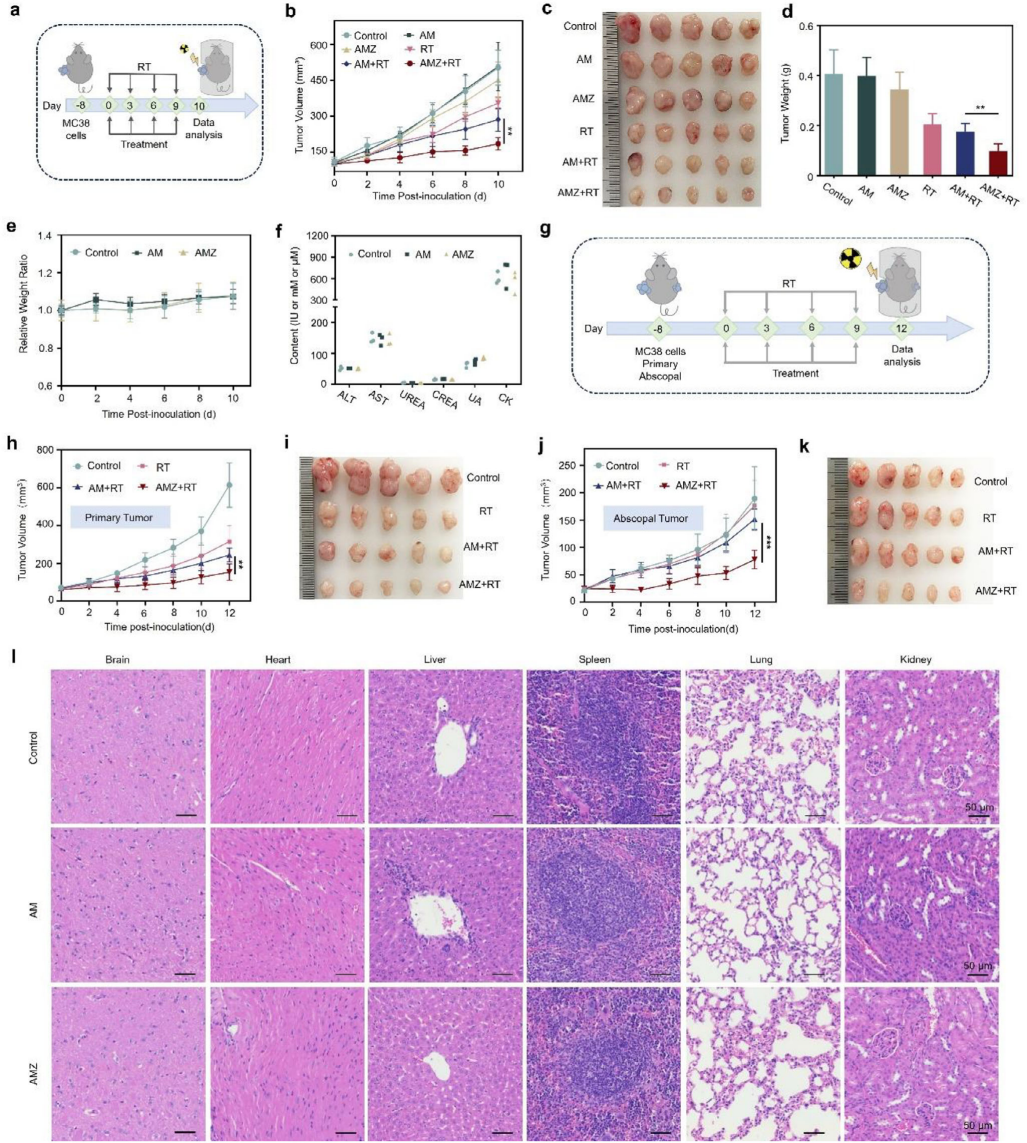
a) Schematic illustration of the treatment process. Only the tumor areas of the mice were irradiated, while the rest of the body was shielded with a lead apron. b) The tumor growth curves, c) isolated tumor images, and d) tumor weight changes (*n* = 5). e) The body weight changes of mice with different treatments (*n* = 5). f) Blood biochemical indexes of mice with different treatments (*n* = 3). g) Schematic illustration of the treatment strategy on the bilateral subcutaneous tumor mice model. Tumor volume and isolated tumor images of primary tumors h,i) and j,k) abscopal tumors (*n* = 5). l) Representative H&E staining images of the main organs of mice with different treatments. Statistical analyses were determined by one‐way ANOVA. ^**^ *p* < 0.01, ^***^ *p* < 0.001.

We subsequently evaluated the in vivo antitumor efficacy of AMZ in tumor‐bearing mice (**Figure** **4**a). As shown in Figure 4b–d, the AM and AMZ groups showed weak tumor growth inhibition compared to the control group. Upon irradiation, the AM+RT group exhibited radiosensitization effects and caused significant tumor regression. In addition, AMZ+RT had the smallest tumor volume and weight, showing the highest tumor inhibition ratio in all groups. This effect was attributed to the combination of AMZ and RT, which not only enhanced radiosensitivity and aggravated the subsequent ICD, but also terminated the immunosuppression of T cells by effectively blocking the interaction between PD‐1 and PD‐L1. In addition, there were no significant changes in body weight (Figure 4e) and blood biochemical indexes among all groups (Figure 4f). These results indicated that the nanoplatform exerted no adverse effects on the physiological functions of the body. The hemolysis test showed that AMZ had low hemolytic activity (Figure S20, Supporting Information), confirming good blood compatibility. Moreover, hematoxylin and eosin (H&E) staining images of major organs revealed no significant side effects from various treatments (Figure 4l), further confirming the safety of AMZ.

### 2.6 AMZ‐Mediated Radio‐Immunotherapy Performance

The ideal tumor treatment strategy should not only achieve primary tumor suppression but also identify and eliminate distant tumors. A bilateral subcutaneous MC38 model was used to investigate the antitumor effects of AMZ in vivo (Figure 4g). Only the AMZ+RT group showed significant inhibitory effects on both primary and distant (abscopal) tumors, while none of the other groups did (Figure 4h–k; Figures S21 and S22, Supporting Information). This may be attributed to the induction of ICD by AMZ+RT and the activation of anti‐tumor immune response, suggesting that AMZ+RT can inhibit the growth of metastatic tumors.

**Figure 5:**
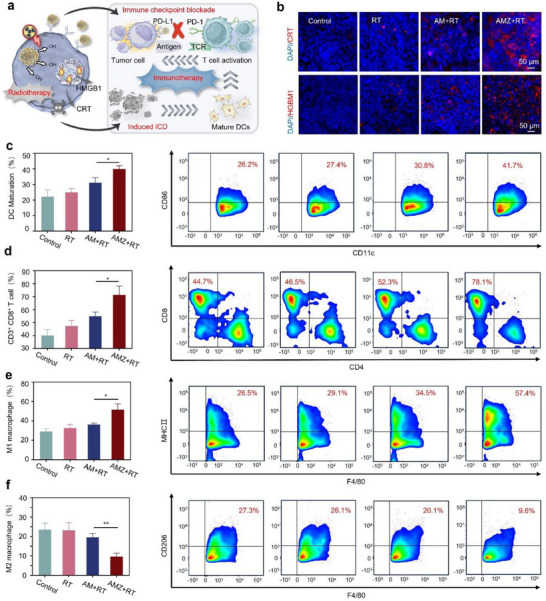
a) Schematic illustration of AMZ‐mediated radio‐immunotherapy. b) Immunofluorescence images of primary tumor slices stained by CRT or HMGB1‐specific antibodies. c) Flow cytometric analysis of mature DC cells (CD11c^+^ CD86^+^), d) CD8^+^ T cell (CD3^+^ CD8^+^) (*n* = 3). e) M1‐TAM (F4/80^+^ MHC II^+^) and f) and M2‐TAM (F4/80^+^ CD206^+^) in abscopal tumors (*n* = 3). Statistical analyses were determined by one‐way ANOVA. ^*^ *p* < 0.05. ^**^ *p* < 0.01.

Subsequently, the antitumor mechanism of AMZ‐mediated radioimmunotherapy was further explored (**Figure** **5**a). We used immunofluorescence to detect the expression of CRT and HMGB1 in primary MC38 tumors after different treatments. As shown in Figure 5b, tumor cells treated with AMZ+RT showed more CRT and HMGB1 red fluorescence signals than the other groups. These results suggested that AMZ can effectively amplify oxidative stress, leading to exposure or release of DAMPs and the induction of ICD. To further investigate the effect of DAMPs exposure or release on tumor DC maturation in vitro, FCM results showed a significant increase in the proportion of mature DC cells in the AMZ+RT group (41.7%) compared to the RT and AM+RT groups (Figure 5c). Meanwhile, the proportion of CD8^+^ T cells of the AMZ+RT group was significantly increased, indicating that AMZ, when combined with RT, can effectively sensitize radiotherapy and intensify subsequent ICD effects (Figure 5d).

AMZ blocking of the PD‐1/PD‐L1 checkpoint enhanced the role of CD8^+^ T cells in immunotherapy. The AMZ+RT group demonstrated the highest increase in the proportion of M1‐TAMs in abscopal tumors (Figure 5e), while the proportion of M2‐TAMs was most significantly decreased as compared to the other groups (Figure 5f). In addition, the IFN‐γ level in the AMZ+RT group increased significantly, further demonstrating the immunotherapeutic efficacy of AMZ (Figure S23, Supporting Information). These results suggested that AMZ‐mediated radioimmunotherapy strategies can promote systemic anti‐tumor immune responses and activate systemic anti‐cancer immunity against distant or metastatic tumors by inducing potent ICDs and PD‐1/PD‐L1 immune checkpoint blocking.

### 2.7 Exploration of Relieving Radiation Enteritis

**Figure 6:**
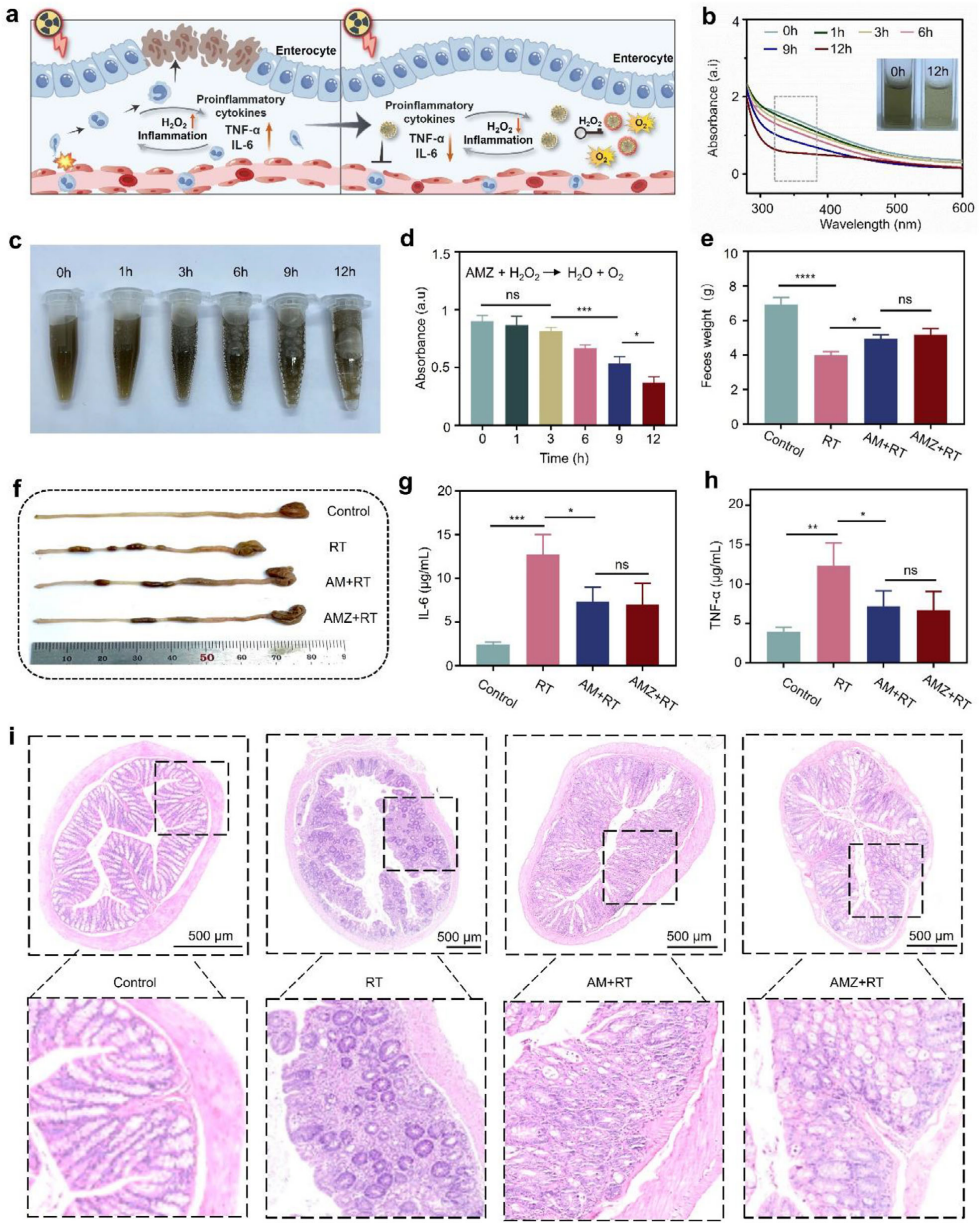
a) Schematic illustration of the mechanism of relieving radiation enteritis. b) UV–vis spectra of AMZ treated with H_2_O_2_ at different times. c) Optical photos of AMZ treated with H_2_O_2_ at different times. d) The H_2_O_2_ content of AMZ treated with H_2_O_2_ at different times (*n* = 3). e) Weight of feces collected from mice after treatment (*n* = 5). f) Representative photographs of the small intestine after dissection showed reduced intestinal obstruction in AMZ+RT. The expression level of g) TNF‐α and h) IL‐6 (*n* = 3). i) H&E staining of colon tissue after treatment (*n* = 3). The scale was 500 µm. Statistical analyses were determined by one‐way ANOVA. ^*^ *p* < 0.05, ^**^ *p* < 0.01, ^***^ *p* < 0.001, ^****^ *p* < 0.0001.

The potential damage to normal intestinal tissues is a major factor limiting the efficacy of RT. Given the remarkable anti‐inflammatory properties exhibited by Ag, the potential of AMZ to relieve radiation enteritis was thoroughly evaluated (**Figure** **6**a). Initially, under a simulated inflammatory environment, the UV–vis spectra of AMZ over time were tracked. As shown in Figure 6b, the intensity of the shoulder peak at ≈350 nm decreased and gradually disappeared, while the color of the solution gradually became lighter, which may be due to the redox reaction of AMZ with H_2_O_2_, leading to the structural degradation of AMZ. The TEM images after 24 h showed that the Ag part of AMZ was selectively etched by H_2_O_2_, and the EDS energy spectrum showed a marked reduction in Ag content, further confirming the H_2_O_2_ scavenging capacity of AMZ (Figures S24 and S25, Supporting Information). Meanwhile, the reaction between AMZ and H_2_O_2_ was accompanied by the generation of bubbles, indicating the consumption of H_2_O_2_ and the generation of O_2_ (Figure 6c). Subsequently, dynamic monitoring revealed a time‐dependent decrease in H_2_O_2_ concentration, accompanied by a corresponding increase in O_2_ production, which validated the significant potential of AMZ in alleviating radiation enteritis (Figure 6d; Figure S26, Supporting Information). Furthermore, to gain in‐depth insights into the H_2_O_2_ decomposition kinetics of AMZ, the Michaelis‐Menten kinetic parameters and maximum reaction rate (V_max_) of AMZ's catalase‐mimetic catalytic process were determined by using an H_2_O_2_‐based reaction system. The results demonstrated that AMZ exhibited relatively slow catalytic kinetics (Figure S27, Supporting Information).

Furthermore, in vivo fluorescence imaging of mice revealed that AMZ effectively accumulated in the intestinal region (Figure S28, Supporting Information). Notably, the reaction between AMZ and H_2_O_2_ progressed relatively slowly, and the delay in intestine metabolism provided favorable conditions for AMZ to alleviate radiation‐induced enteritis, which has important practical implications for its in vivo application. Subsequently, a mice enteritis model was constructed to further verify the alleviating effect of AMZ on radiation enteritis. Mice in the radiation group all showed colitis symptoms, including decreased appetite and constipation, primarily due to rapid proliferative intestinal cell death and acute inflammatory response in the lamina propria. Compared to normal mice, the RT group had significantly lower fecal weight while the AMZ+RT group had improved constipation, indicating the relief of AMZ on radiation enteritis (Figure 6e). In addition, we collected intestinal tissue from the mice after fasting for 24 h, and the accumulation of feces confirmed the radiation‐induced colon tissue damage. As shown in Figure 6f, the AMZ+RT group showed less severe intestinal obstruction and longer colon length compared to the RT group, indicating that AMZ significantly alleviated radiation enteritis by scavenging H_2_O_2_ in the inflamed area (Figure 6f). In addition, analysis of H_2_O_2_ levels in intestine tissues revealed a notable reduction of ≈41.8% within the AMZ+RT group compared to the RT group. This further confirmed that AMZ can effectively scavenge H_2_O_2_, thereby alleviating radiation enteritis (Figure S29, Supporting Information).

H&E staining of intestinal tissues showed that the AMZ+RT group had a slight protective effect on colonic mucosa, inhibiting the destruction of epithelial cells, the abnormality of crypt structure, and the infiltration of immune cells, thus reducing the damage of intestinal tissue (Figure 6i). Although the exact pathogenesis of radiation enteritis remained unclear, immune and inflammatory responses certainly played a crucial role. As shown in Figure 6g,h, the RT group induced a strong inflammatory response that promoted the expression of proinflammatory cytokines such as tumor necrosis factor (TNF‐α) and interleukin‐6 (IL‐6). FCM results showed that M1‐TAMs were significantly lower in the AMZ+RT group than in the RT group, while M2‐TAMs were significantly higher, further verifying the anti‐inflammatory effect of AMZ (Figure S30, Supporting Information). In contrast, the AMZ+RT group significantly reduced the expression of these proinflammatory cytokines, indicating the great anti‐inflammatory effects of AMZ. These results demonstrated that AMZ effectively reduced radiation‐induced colon damage, demonstrating the great potential of the nanoplatform in radiosensitization and radioprotection.

## 3 Conclusion

In summary, a Janus structured nanoplatform AMZ was dexterously developed. Upon X‐ray exposure, the nanoplatform can achieve abundant energy deposition within tumors. Subsequently, because of the difference in Schottky barriers, radiation‐induced electrons transferred from Ag_2_S to Ag moiety enable effective electron–hole pair separation, which greatly improved the radiocatalysis efficiency to decompose H_2_O_2_ into •OH. Moreover, radiosensitization of AMZ also significantly induced ICD, stimulated antigen presentation, and enhanced anti‐tumor immune response. Z_PD‐L1_ modification not only endowed AMZ with stronger tumor targeting, but also effectively blocked PD‐1/PD‐L1 interaction, terminating T cell immunosuppression and achieving ICD‐PD‐1/PD‐L1 synergistic immunotherapy. Interestingly, AMZ effectively decreased the overexpressed H_2_O_2_ level of the inflammatory intestinal tissues induced by X‐ray exposure, achieving the alleviation of radiation enteritis. This effective management ultimately achieved a refined radiotherapy strategy characterized by “toxicity reduction and efficacy enhancement”. Overall, AMZ offered a promising treatment strategy to increase radiosensitivity while simultaneously providing radioprotection benefits.

## 4 Experimental Section

Details regarding the experimental procedures can be found in the Supporting Information. Data were analyzed using Graphpad Prism 7.0 and Origin 8.0 software. The results were expressed in terms of mean values, accompanied by their standard deviations. Depending on the suitability of the data, either one‐way ANOVA or two‐way ANOVA was employed for statistical evaluation. Statistical analyses were determined when the *p*‐value was less than 0.05, denoted as follows: ns for *p*‐values greater than 0.05, a single asterisk (^*^) for *p*‐values less than 0.05, double asterisks (^**^) for *p*‐values less than 0.01, triple asterisks (^***^) for *p*‐values less than 0.001, and quadruple asterisks (^****^) for *p*‐values less than 0.0001. Animals were cared for and maintained under the Guidelines of Laboratory Animals of Shandong First Medical University and approved by the Animal Ethics Committee of Shandong First Medical University (202306050501).

## Conflict of Interest

The authors declare no conflict of interest.

## Supporting information



Supporting Information

## Data Availability

All data generated or analyzed in this study are available upon reasonable requests to the lead contact if included or visualized in this published article.
