# Supplementary material for: Convertible ROS Nanocatalyst/Eliminator for Enhancing Tumor Radio‐Immunotherapy and Relieving Radiation Enteritis
Source: Adv Sci (Weinh). 2025 Jun 10;12(33):e03602. doi: 10.1002/advs.202503602 (PMC12412606; doi:10.1002/advs.202503602)
Supplement: Supplementary file 1 — Supporting Information [file ADVS-12-e03602-s001.docx]

**Supplementary Information**

**Convertible ROS Nanocatalyst/Eliminator for Enhancing Tumor Radio-Immunotherapy and Relieving Radiation Enteritis**

Mengmeng Zhang,^1^ Xinyu Zhang,^2^ Chenyu Wang,^2^ Mengyao Mu,^2^ Ke Ren,^2^ Nengyi Ni,^3^ Li Xian Yip,^3^ Kai Guo,^2^ Xiaoyu Hu,^1^ Feifei Teng,^1^ David Tai Leong,^3^ Qing Fan,^1,^* Xiao Sun^1,^*

^1^ Department of Pharmacy, Shandong Cancer Hospital and Institute, Shandong First Medical University & Shandong Academy of Medical Sciences, Jinan 250117, China

^2^ Medical Science and Technology Innovation Center, Tumor Research and Therapy Center, Shandong Provincial Hospital Affiliated to Shandong First Medical University, Jinan 250021, China

^3^ Department of Chemical and Biomolecular Engineering, National University of Singapore, Singapore 117585, Singapore

*Corresponding authors

E-mail: sunxiao@sdfmu.edu.cn (X. Sun); fanqing@sdfmu.edu.cn (Q. Fan)

**Materials and Methods**

**Materials**

All chemical reagents were used directly without further purification. Ag(acac)_2_, trisodium citrate, polyethyleneimine (PEI), thioacetamide, triethanolamine, SH-PEG-NH_2_, NHS-DOTA-ester, Manganese (II) chloride (MnCl_2_), NHS-FEG2000-SH, 3',5,5'-Tetramethylbenzidine (TMB) were purchased from Aladdin Chemical Co. Ltd (Shanghai, China). Fetal bovine serum (FBS) was purchased from lonsera (USA). Dulbecco's modified eagle medium (DMEM), Phosphate-buffered saline (PBS) and penicillin-streptomycin, H33342 trihydrochloride (Hoechst 33342), pancreatic enzymes, Membrane and Cytosol Protein Extraction Kit were purchased from Beyotime Institute of Biotechnology (Haimen, China). BCA protein assay kit, cell lysis buffer for western Blotting and IP, primary antibody dilution buffer and other commonly used buffers were purchased from Aladdin Chemical Co. Ltd (Shanghai, China). Cy5.5-NHS was purchased from Shanghai Maokang biotechnology Co. Ltd. Mitochondrial Membrane Potential Assay Kit with JC-1 was purchased from Solarbio Science & Technology Co., Ltd (Beijing, China). Annexin V-FITC/7-AAD Apoptosis Detection Kit was purchased from Dalian Meilun Biotechnology Co., Ltd. (Dalian, China).

**Characterization**

The morphology and elemental distribution of the particles were observed by TEM. Crystal structure measurements were performed on XRD (MiniFlex 600, Rigaku, Japan). The composition and structure of the sample were measured on XPS (ESCALAB 250 Xi, Thermo Scientific, USA) and FTIR spectrometer (Tensor 27, Brookhaven, USA). The particle size distribution and Zeta potential were measured using a DLS detector (NanoBrook 90 Plus Pals, Brookhaven, USA). Metal content was quantified on an ICP-OES (G8018A, Agilent, USA).

**Synthesis of Ag/Ag_2_S**

Firstly, Ag(acac)_2_ (0.06 mmol) and trisodium citrate (0.2 mmol) were dissolved in 15mL ethylene glycol, followed by addition of 150 mg polyethyleneimine (PEI) under vigorous stirring for 2 h. Then, the thioacetamide (0.05 M, 1.5 mL) and 0.5 mL triethanolamine were consecutively added and stirred for 10min. Finally, the resulting mixture was transferred to a microwave synthesizer and reacted at 200°C for 1 h to prepare Ag/Ag_2_S.

**Synthesis of Ag/Ag_2_S-Mn (AM)**

First, 2 mg Ag/Ag_2_S and 3 mg SH-PEG-NH2 were dissolved in 4 mL H_2_O, stirred vigorously at 4℃ for 6 h, centrifuged and redispersed in 4 mL H_2_O. Then, after adjusting the pH of the solution, 3 mg NHS-DOTA was added and stirred for 0.5 h. Finally, 0.4 mg MnCl_2_ was added to the resulting mixture and stirred for 6 h to prepare AM.

**Synthesis of Z_PD-L1_ affibody**

Based on the amino acid sequence of M1 affibody, the coding sequence for Z_PD-L1_ was computationally optimized and modified by incorporating a cysteine residue at the C-terminal end to enable disulfide-mediated dimerization. The synthetic coding gene, commercially synthesized by GenScript (Nanjing), was subcloned into the BamHI/SalI restriction sites of the pQE-30 vector, generating the pQE30-Z_PD-L1_ expression construct. This plasmid features an N-terminal hexahistidine tag to facilitate immobilized metal affinity chromatography. Following transformation into E. coli M15 cells, protein expression was induced with 0.1 mM IPTG at 28°C for 16 h. Harvested bacterial pellets were resuspended in ice-cold lysis buffer (50 mM phosphate, 300 mM NaCl, 20 mM imidazole, pH 8.0) and subjected to ultrasonic disruption. The clarified lysate was incubated with Ni-NTA resin (GenScript) for target protein purification. Post-purification, the Z_PD-L1_ solution underwent buffer exchange into PBS (137 mM NaCl, 2.7 mM KCl, 4.3 mM Na_2_HPO_4_, 1.4 mM KH_2_PO_4_, pH 7.4) through overnight dialysis at 4°C, with protein quantification performed using the Bradford method. The molecular weight and aggregation forms of Z_PD-L1_ were analyzed by SDS−PAGE with the absence (natural conditions) or presence (reductive conditions) of β-mercaptoethanol (2-ME).

**Synthesis of Ag/Ag_2_S-Mn-Z_PD-L1_ (AMZ)**

First, the prepared Z_PD-L1_ was mixed with NHS-PEG2000-SH (mass ratio = 20 μg: 1 mg) under vigorous stirring at 4 ℃ for 6 h. Then, AM was added to the mixture and stirred for 0.5 h, and finally the resulting AMZ was washed 3 times before further use.

**Electrochemical measurements**

The electrochemical measurements were performed on a standard three electrode electrochemical workstation (CHI 660D) at 25 °C. A Pt wire and an Ag/AgCl electrode were selected as the counter electrode and reference electrode, respectively. Before measurement, the GCEs were first polished with 0.05 μm alumina powder carefully, and then washed with Milli-Q water and ethanol under ultrasonication, followed by drying at room temperature. The working electrodes were prepared by drop-coating catalysts ink (including Ag, Ag_2_S, and Ag/Ag_2_S) onto the glass carbon electrode (GCE). Cyclic voltammogram (CV) and EIS Nyquist curves of the catalysts (Ag, Ag_2_S, and Ag/Ag_2_S) were recorded in a 0.5 M KOH solution at a scan rate of 20 mV s^−1^, using X-rays as the excitation light source and focusing on the working electrode.

**•OH detection**

TMB assay was used to determine that Ag/Ag_2_S catalyzes the conversion of H_2_O_2_ to •OH. Briefly, H_2_O_2_ (100 μM) and TMB (1 mM) were mixed with AMZ under X-ray irradiation (6 Gy). Then, the UV-Vis absorption spectra of the mixture were detected.

**Cell uptake**

MC38 cells were inoculated in the 6-well plates, treated with Cy5.5-labeled AM, AMZ, Z_PD-L1_+AMZ for 4 h. Then, the cells were collected and analyzed by FCM. After the same administration operation as described above, the cells were observed using a CLSM to obtain fluorescence images.

**Biocompatibility assay**

HUVEC in logarithmic growth phase were taken and inoculated in 96-well plates, and cultured in DMEM at 37 °C with 5% CO_2_. After that, the cells were treated by AMZ with different concentrations for 24 h.

**Cytotoxicity assay**

MC38 were inoculated in the 6-well plates, treated with PBS, AM or AMZ (40 μg/mL of Ag) and incubated for 4 h. Then the cells were irradiated with a dose of 6 Gy, while tumor cells that did not receive radiation served as the control. After radiation, MC38 cells were cultured continuously for another 24 h and cell viability were determined by MTT assay.

**Cell apoptosis**

MC38 cells were inoculated in the 6-well plates. Then, they were treated with PBS, AM or AMZ (40 μg/mL of Ag) and incubated for 4 h. Following treatment, the cells were irradiated with a dose of 6 Gy, while tumor cells that did not receive radiation served as the control. After radiation, MC38 cells were cultured continuously for another 24 h, digested and collected, stained with Annexin V-FITC and 7-AAD probe, and the percentage of apoptosis was analyzed by FCM.

**Clonogenic assay**

MC38 cells were inoculated in the 6-well plates. Then, they were treated with PBS, AM or AMZ (40 μg/mL of Ag) and incubated for 4 h. Following treatment, the cells were irradiated with a dose of 6 Gy, while tumor cells that did not receive radiation served as the control. The cells were then incubated for another 7 days, fixed with methanol: acetic acid (3:1) and stained with crystal violet. Finally, the number of colonies was counted.

**Scratch test**

MC38 cells were inoculated in migration chamber for overnight. The migration chambers were then removed, and treated with PBS, AM or AMZ (40 μg/mL of Ag) for co-incubation. Following treatment, the cells were irradiated with a dose of 6 Gy, while tumor cells that did not receive radiation served as the control. Scratch healing was observed at 0 and 48 h.

**Intracellular •OH detection**

MC38 cells were inoculated in the 6-well plates, Then, they were treated with PBS, AM or AMZ (40 μg/mL of Ag) and incubated for 4 h. Following treatment, the cells were irradiated with a dose of 6 Gy, while tumor cells that did not receive radiation served as the control. The cells were incubated with Hoechst and DCFH-DA fluorescent probes in dark conditions for 30 min, and the fluorescence intensity was observed by CLSM.

**Mitochondrial membrane potential evaluation**

MC38 cells were inoculated in the 6-well plates. Then, they were treated with PBS, AM or AMZ (40 μg/mL of Ag) and incubated for 4 h. Following treatment, the cells were irradiated with a dose of 6 Gy, while tumor cells that did not receive radiation served as the control. JC-1 mitochondrial membrane potential fluorescence probe was added and incubated at 37 ℃ for 30 min. The membrane potential was observed by CLSM.

**Detection of CRT exposure**

MC38 cells were inoculated in the 6-well plates. Then, they were treated with PBS, AM or AMZ (40 μg/mL of Ag) and incubated for 4 h. Following treatment, the cells were irradiated with a dose of 6 Gy, while tumor cells that did not receive radiation served as the control. MC38 cells were then fixed, permeabilized, exposed to blocking buffer, and stained with mouse anti-CRT. After several washes, cells were incubated with secondary antibodies. Cell nuclei were stained with Hoechst. The intracellular CRT were analyzed by CLSM.

**Detection of HMGB1 release**

HMGB1 concentrations in the cytoplasm of MC38 cells following the indicated treatments were measured by ELISA kit, according to the manufacturer’s protocol. Luminescence and absorbance were measured by using microplate reader.

**Western blot**

MC38 cells were inoculated in 6-well plates. Then, they were treated with PBS, AM or AMZ (40 μg/mL of Ag) and incubated for 4 h. Following treatment, the cells were irradiated with a dose of 6 Gy, while tumor cells that did not receive radiation served as the control. After radiation, MC38 cells were cultured continuously for another 24 h. The RIPA buffer was added with PMSF to lyse the cells and extract the protein. The protein was analyzed using SDS-PAGE and transferred to PVDF membrane for imaging. After being sealed with 5% skim milk, it was sequentially incubated with Bcl-2 and Bax labeled goat anti-mouse secondary antibody, and finally enhanced chemiluminescence ECL was developed.

**Fluorescent tissue distribution *in vivo***

For *in vivo* fluorescence imaging, MC38 tumor-bearing mice were intravenously injected with AM-Cy5.5 and AMZ-Cy5.5 (0.1 mL, 2 mg/kg in saline), and scan images were acquired using small animal *in vivo* imaging system (IVIS®Lumina Series III, PerkinElmer, USA) at 1, 2 and 4 h. Mice were dissected at 4 h to attain each major organ for fluorescence imaging.

**Mice metabolism analysis**

All animal experiments were approved by Bioethics Committee of Shandong First Medical University (202306050501). Mice were intravenously injected with AMZ (0.1 mL, 2 mg/kg in saline), and feces were collected at 24 h, 48 h,72 h, 96 h,120 h by using ICP-OES to detect the Ag^+^ concentration.

**MRI *in vitro* and *in vivo***

For *in vitro* MRI, AMZ solutions with different concentrations were scanned by MR to detect T1 relaxation rate. For cell MRI, MC38 cells treated with AM and AMZ were scanned by 9.4 T MR. For in vivo MRI, MC38 tumor-bearing mice were intravenously injected with AM and AMZ (0.1 mL, 2 mg/kg in saline), and scan images by 9.4 T MR were obtained at 0,0.25, 0.5, 1, 1.5, 2 h after injection (Bruker, TR = 6.0 ms, TE = 800 ms, slice thickness = 0.8 mm).

**Hemolysis test**

Mice blood was centrifuged and washed to obtain red blood cells (RBCs) and resuspended in PBS. AMZ was added to the erythrocyte suspension with various concentrations. Incubate them at 37 ℃ for 3 h, centrifuge and take photos. The hemolysis rate was determined and calculated.

**Exploration of tumor inhibition in vivo**

MC38 cells were dispersed into serum and injected subcutaneously via the left side to establish a C57BL/6 mice model. After the tumor reached an initial size of approximately 100 mm^3^, the mice were randomly divided into 6 groups (*n=5*), and injected with PBS, AM or AMZ. After 1 h, the tumors were irradiated (6 Gy), while tumor that did not receive radiation served as the control. During the treatment period, the drug and RT were given once every 3 days for a total of 4 times. Total body weight and tumor size were recorded every 2 days. After 10 days, the mice were sacrificed, the tumors were extracted, weighed, and photographed. The tumor volume (V) is determined by V=a × b^2^/2 calculation, where "a" and "b" are the longest and shortest diameters of the tumor, respectively.

**Abscopal effect**

MC38 cells were subcutaneously injected into the left and right lower abdomen of mice to form primary and abscopal tumors, respectively. After 8 days, the mice were randomly divided into PBS, RT, AM+RT and AMZ+RT groups (*n=5*). RT was administered 1 hour after the intravenous injection of the nanomedicine, with only the primary tumor receiving radiation treatment. During the treatment period, the drug and RT was given once every 3 days for a total of 4 times. Total body weight and tumor size were recorded every 2 days. After 12 days, the mice were sacrificed, the tumors were extracted, weighed, and photographed.

**Immunotherapy *in vivo***

Flow cytometry was used to determine the percentages of mature DC cells (CD11c^+^ CD86^+^), CD8^+^ T-cell (CD3^+^ CD8^+^), M1-phenotype macrophages (F4/80^+^ MHCII^+^), and M2-phenotype macrophages (F4/80^+^ CD206^+^) in abscopal tumor tissues.

***In vivo* safety**

At the end of treatment, whole blood was collected in each group by removing the eyeballs. The blood specimens were allowed to stand overnight at 4 °C, then centrifuged at 3000 r·min^-1^ for 15 min, and the supernatants were harvested for biochemical analysis. This analysis included liver-related indexes: alanine aminotransferase (ALT), aspartate aminotransferase (AST); renal-related indexes: urinary uric acid (UREA), creatinine (CREA), uric acid (UA); and cardiac enzyme profiles: creatine kinase (CK), and lactate dehydrogenase (LDH). At the end of the treatment, major organs of mice (heart, liver, spleen, lungs, kidneys, and brain) were taken and sectioned for H&E staining to observe the pathological changes.

**H_2_O_2_-response of AMZ**

To explore the influence of H_2_O_2_ on the structure of AMZ, H_2_O_2_ (100 μM) was added into the AMZ (0.1 mg/mL) solution, and the reaction process was monitored by UV-vis at different times.

**Detection of O_2_ Concentration**

To visually observe the gas produced during the reaction process, H_2_O_2_ (1 mM) was added to the AMZ (1 mg/mL) solution, and the bubble formation was recorded using a digital camera at different times. Meanwhile, the dissolved O_2_ concentration of the mixed solution was monitored using a dissolved oxygen meter.

**Detection of H_2_O_2_ Level**

H_2_O_2_ (1 mM) was added to the AMZ (1 mg/mL) solution, and the concentration of H_2_O_2_ in the reaction system was measured using the H_2_O_2_ kit at different times.

**Construction of enteritis model**

The mice were randomly divided into PBS group, RT group, AM+RT group and AMZ+RT group (n=5). After 1 hour of intravenous injection of nanomedicine, mice were irradiated with 10 Gy X-ray irradiation to the whole abdominal area. After 6 days, intestinal anatomy and histopathology were observed to evaluate the relieving effect of AMZ on radiation enteritis.

**Statistical analysis**

Data were analyzed using Graphpad Prism 7.0 and Origin 8.0 software. The results were expressed in terms of mean values, accompanied by their standard deviations. Depending on the suitability of the data, either one-way ANOVA or two-way ANOVA was employed for statistical evaluation. Statistical analyses were determined when the P-value was less than 0.05, denoted as follows: ns for P-values greater than 0.05, a single asterisk (*) for P-values less than 0.05, double asterisks (**) for P-values less than 0.01, triple asterisks (***) for P-values less than 0.001, and quadruple asterisks (****) for P-values less than 0.0001.


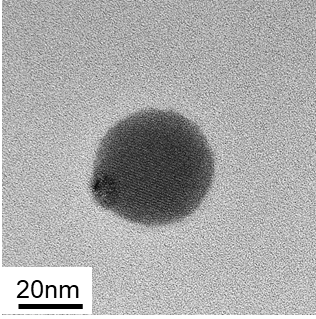


Figure S1. TEM image of AMZ.


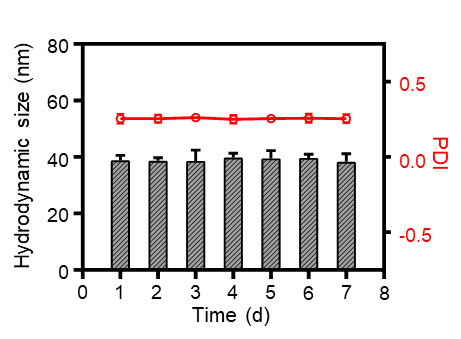


Figure S2. Hydrodynamic size of AMZ within 7 days.


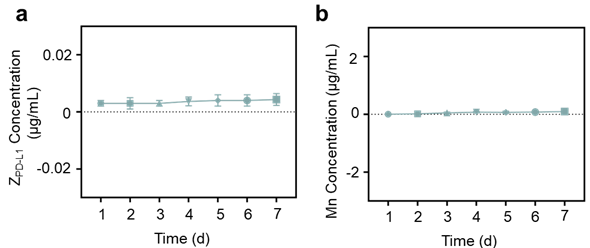


Figure S3. The concentration of Z_PD-L1_ (a) and Mn^2⁺^ (b) within 7 days.


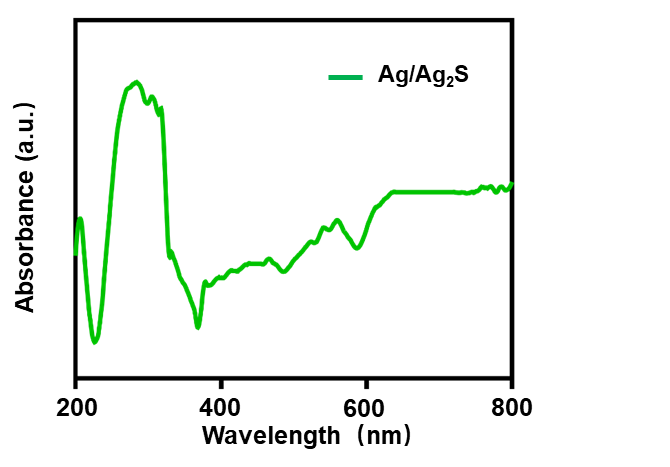


Figure S4. The UV-vis spectrum of Ag/Ag_2_S.


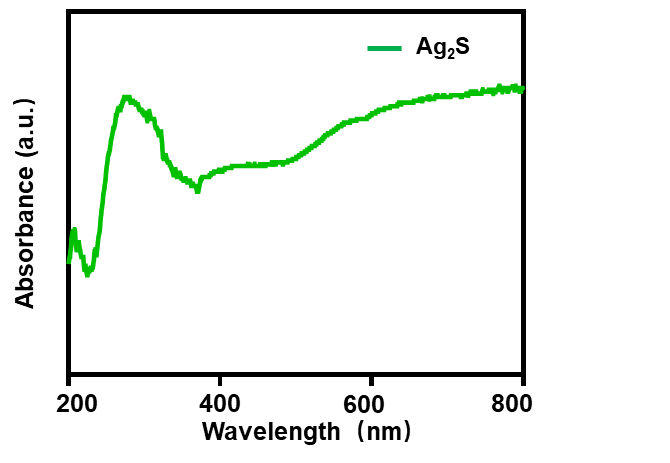


Figure S5. The UV-vis spectrum of Ag_2_S.


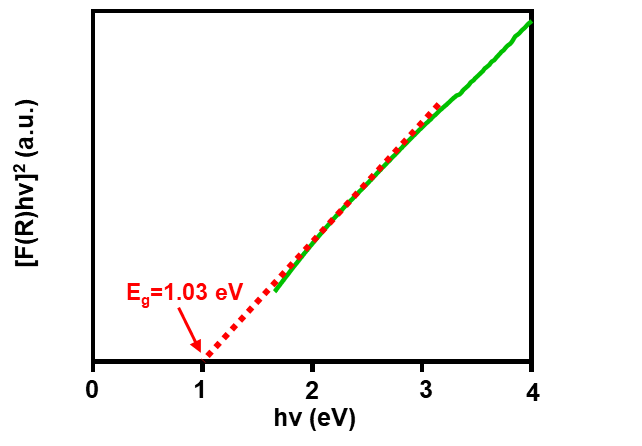


Figure S6. Energy band gap of Ag_2_S.


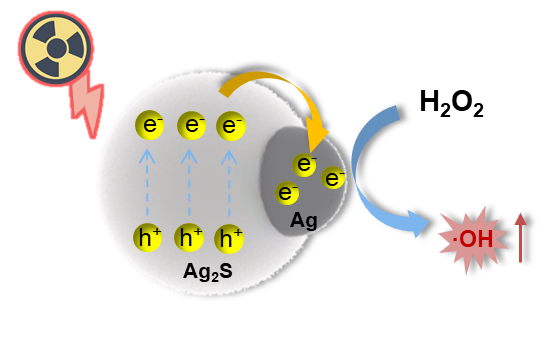


Figure S7. X-rays induce AMZ to catalyze the generation of •OH from H_2_O_2_.


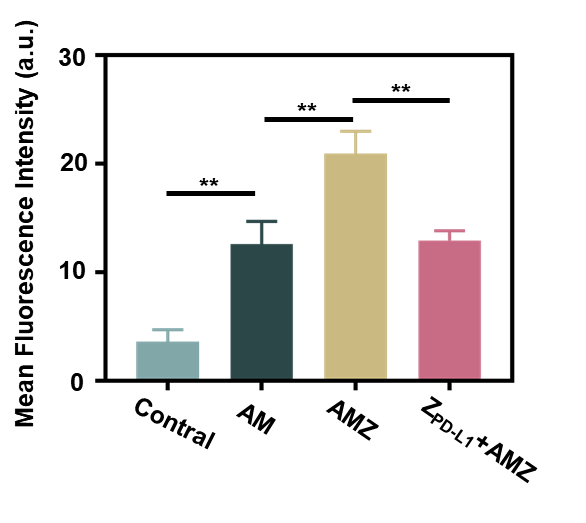


Figure S8. CLSM fluorescence semi-quantitative analysis of cellular uptake. Statistical analyses were determined by one-way ANOVA (*n = 3*). **P < 0.01.


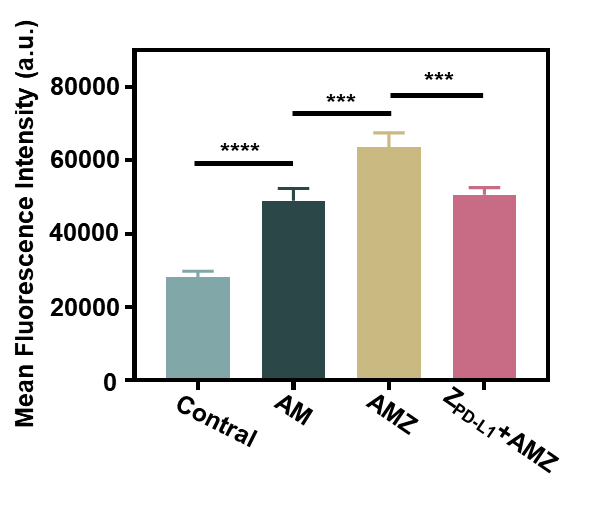


Figure S9. FCM semi-quantitative analysis of cell uptake. Statistical analyses were determined by one-way ANOVA (*n = 3*). ***P < 0.001, ****P < 0.0001.


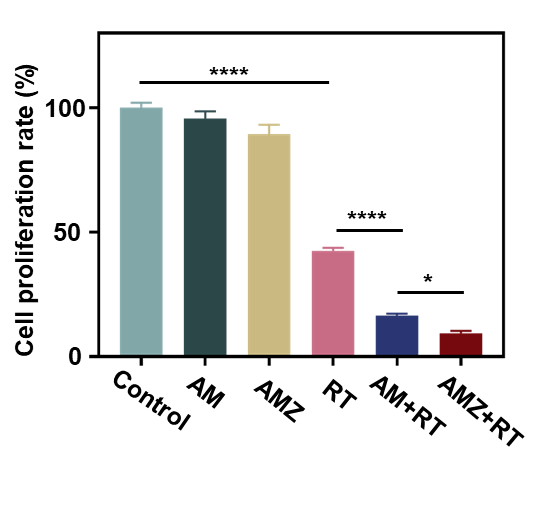


Figure S10. Clone formation images quantitative analysis of MC38 cells with different treatments. Statistical analyses were determined by one-way ANOVA (*n = 3*). *P < 0.05, ****P < 0.0001.


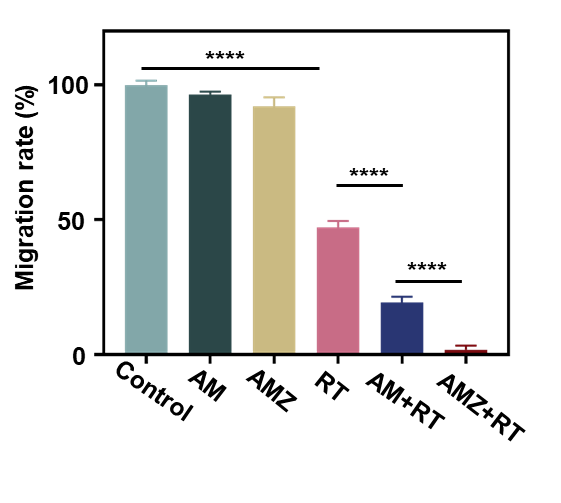


Figure S11. Wound healing images quantitative analysis of MC38 cells with different treatments. Statistical analyses were determined by one-way ANOVA (*n = 3*). ****P < 0.0001.


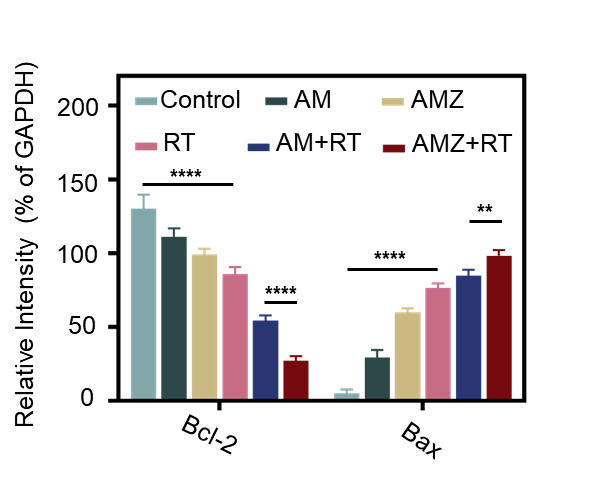


Figure S12. WB semi-quantitative analysis of Bcl-2 and Bax of MC38 cells. Statistical analyses were determined by two-way ANOVA (*n = 3*). **P < 0.01, ****P < 0.0001.


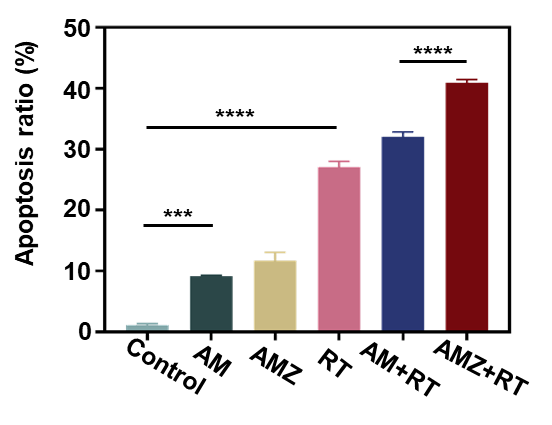


Figure S13. FCM semi-quantitative analysis of apoptosis in MC38 cells with different treatments. Statistical analyses were determined by one-way ANOVA (*n = 3*). ***P < 0.001, ****P < 0.0001.


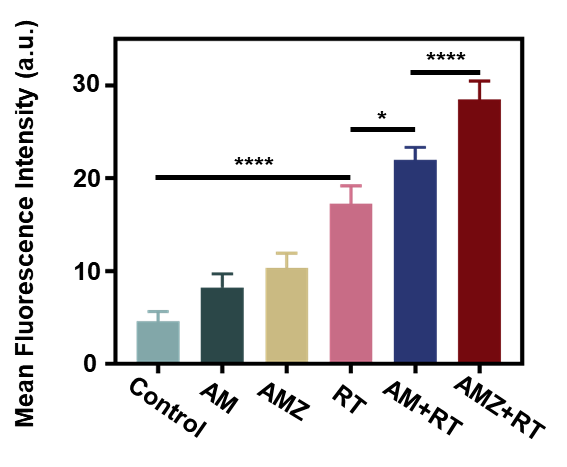


Figure S14. Fluorescence semi-quantitative analysis of MC38 cells incubated with DCFH-DA as fluorescent probe. Statistical analyses were determined by one-way ANOVA (*n = 3*). *P < 0.05, ****P < 0.0001.


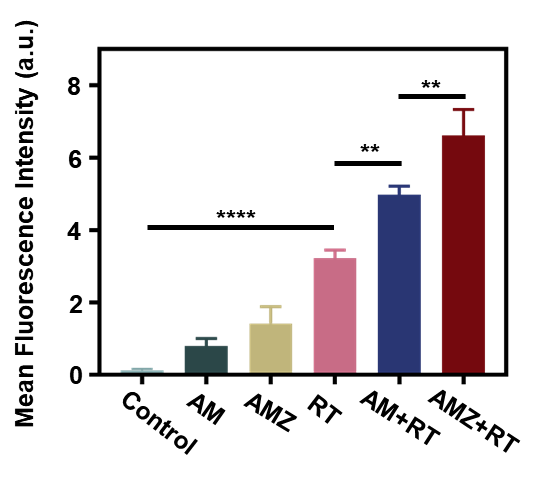


Figure S15. Fluorescence semi-quantitative analysis of MC38 cells incubated with γ-H_2_AX as fluorescent probe. Statistical analyses were determined by one-way ANOVA (*n = 3*). **P < 0.01, ****P < 0.0001.


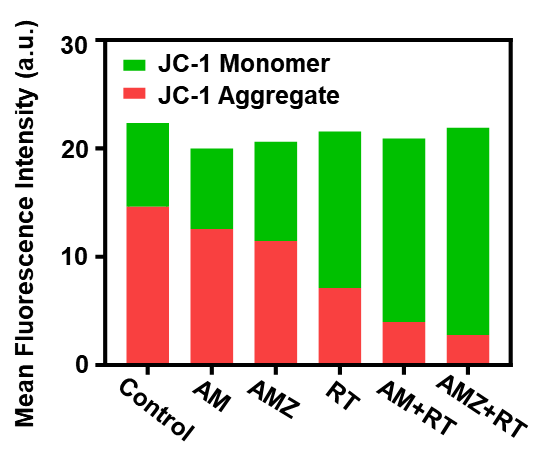


Figure S16. Fluorescence semi-quantitative analysis of MC38 cells incubated with JC-1 as fluorescent probe.


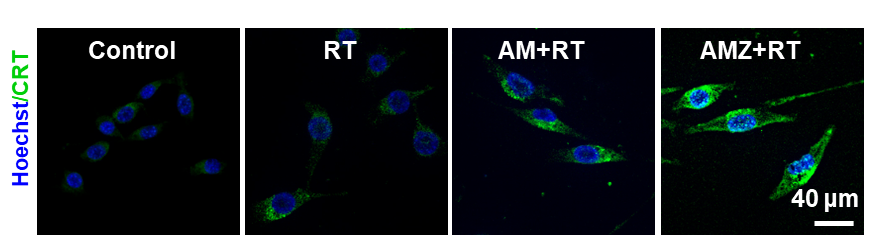


Figure S17. CLSM images of primary tumor slices stained by CRT-specific antibodies.


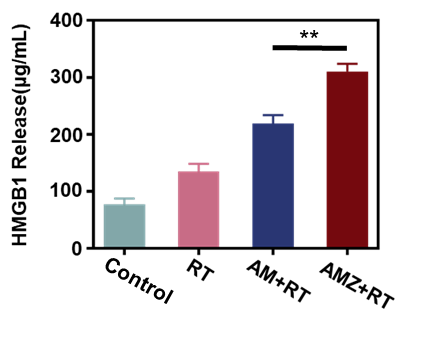


Figure S18. Elisa detection of HMGB1 release of mice after different treatments. Statistical analyses were determined by one-way ANOVA (*n = 3*). **P < 0.01.


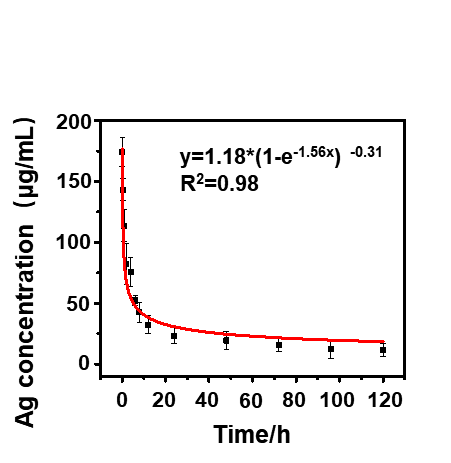


Figure S19. Pharmacokinetics after caudal intravenous administration of AMZ (*n=3*).


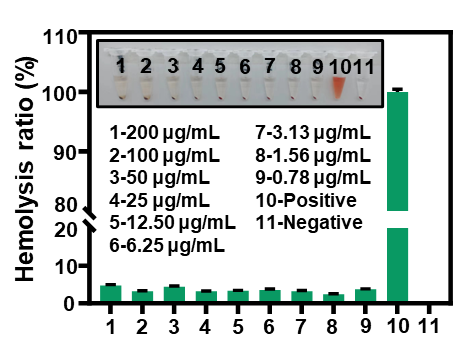


Figure S20. Hemolysis picture and hemolysis rate of AMZ (*n=3*).


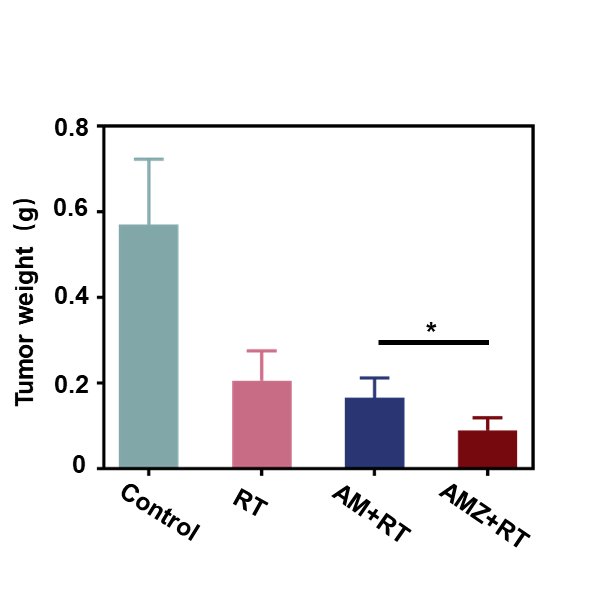


Figure S21. The tumor weight changes of primary tumors. Statistical analyses were determined by one-way ANOVA (*n = 5*). *P < 0.05.


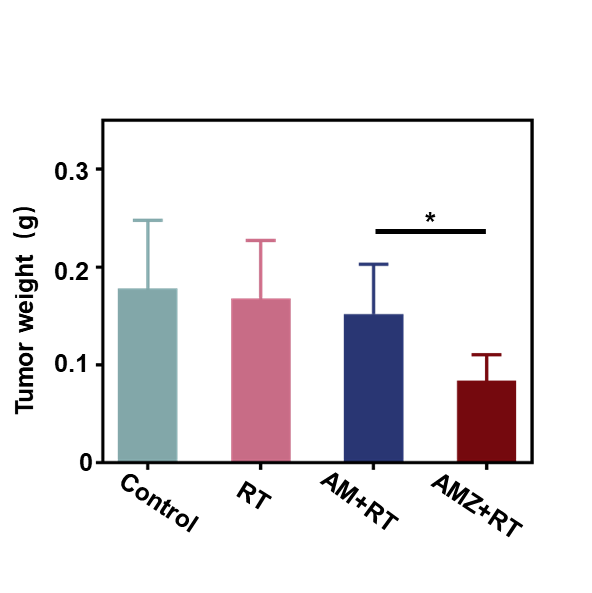


Figure S22. The tumor weight changes of abscopal tumors. Statistical analyses were determined by one-way ANOVA (*n = 5*). *P < 0.05.


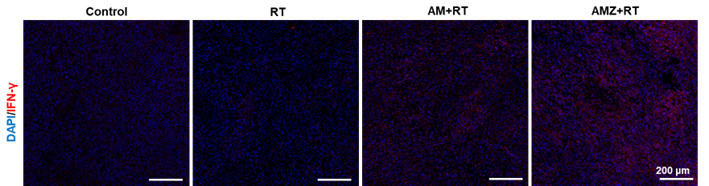


Figure S23. Immunofluorescence images of IFN-γ expression in abscopal tumor after different treatments.


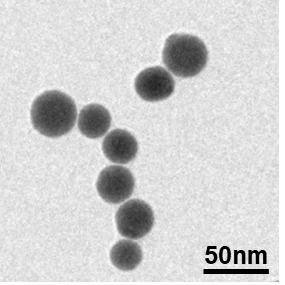


Figure S24. TEM image of AMZ treated with H_2_O_2_ after 24h.


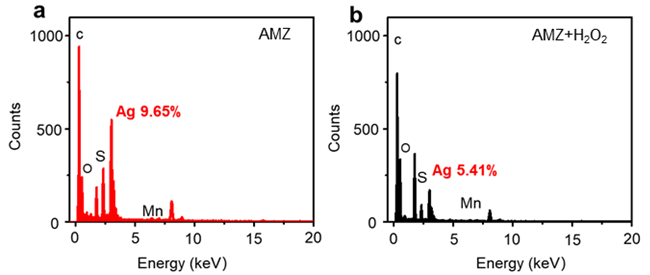


Figure S25. EDS energy spectrum of AMZ before (a) and after (b) H_2_O_2_ treatment.


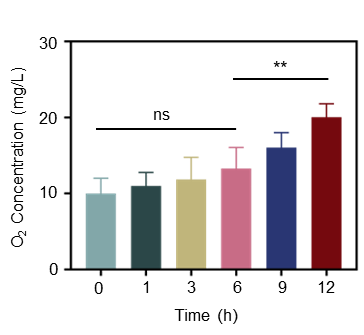


Figure S26. The O_2_ concentration of AMZ treated with H_2_O_2_ at different times. Statistical analyses were determined by one-way ANOVA. **P < 0.01.


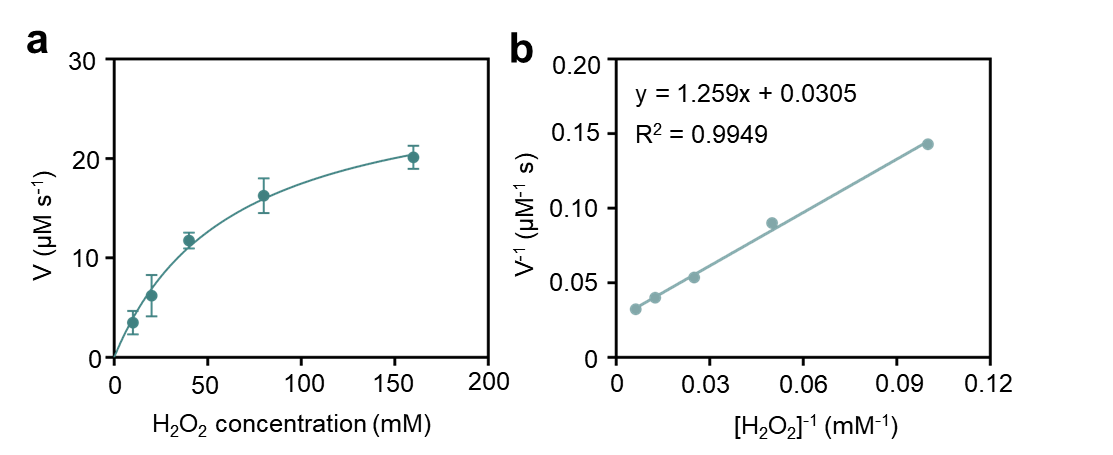


Figure S27. (a) Michaelis-Menten kinetics curve and (b) Lineweaver−Burk plot of CAT-like activity of AMZ.


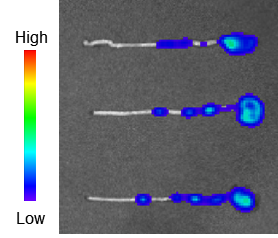


Figure S28. Fluorescence images of isolated intestines after 4 h administration (*n=3*).


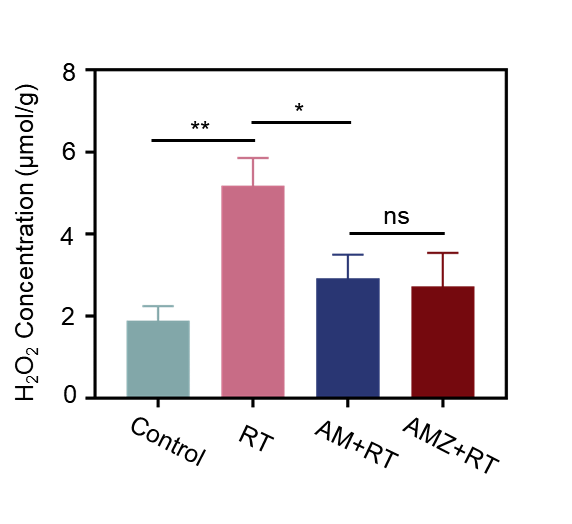


Figure S29. The content of H_2_O_2_ in intestinal tissues after different treatments. Statistical analyses were determined by one-way ANOVA. *p < 0.05, **P < 0.01.


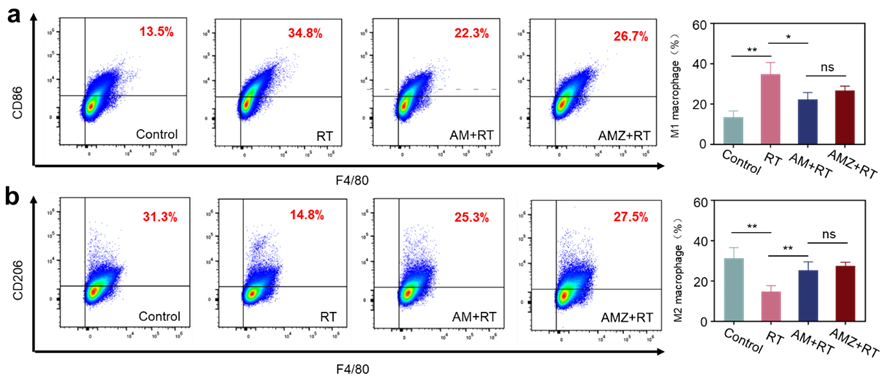


Figure S30. Flow cytometric analysis of (a) M1-TAM (F4/80^+^ CD86) and (b) and M2-TAM (F4/80^+^ CD206^+^) in intestinal tissues (*n=3*). Statistical analyses were determined by one-way ANOVA. *p < 0.05, **p < 0.01.
